# Photosynthetic Responses of Canola and Wheat to Elevated Levels of CO_2_, O_3_ and Water Deficit in Open-Top Chambers

**DOI:** 10.3390/plants8060171

**Published:** 2019-06-12

**Authors:** Bheki G. Maliba, Prabhu M. Inbaraj, Jacques M. Berner

**Affiliations:** 1 Unit for Environmental Sciences and Management, North-West University, Potchefstroom 2520, South Africa; prabhu.inbaraj@gmail.com (P.M.I.); Jacques.berner@nwu.ac.za (J.M.B.); 2 Eskom Research, Testing and Development, Cleveland 2022, South Africa; 3 Department of Chemistry, School of Basic Sciences, Manipal University Jaipur, Jaipur 303007, India

**Keywords:** biomass, canola, drought, elevated CO_2_, JIP-test, open-top chamber, ozone, wheat

## Abstract

The effects of elevated CO_2_ (700 ppm) and O_3_ (80 ppb) alone and in combination on the photosynthetic efficiency of canola and wheat plants were investigated in open-top chambers (OTCs). The plants were fumigated for four weeks under well-watered and water-stressed (water deficit) conditions. The fast chlorophyll *a* fluorescence transients were measured after 2 and 4 weeks of fumigation, as well as in control plants, and analyzed by the JIP-test, which is a non-destructive, non-invasive, informative, very fast and inexpensive technique used to evaluate the changes in photosynthetic efficiency. Biomass measurements were taken only after 4 weeks of fumigation. The performance index (PI_total_), an overall parameter calculated from the JIP-test formulae, was reduced by elevated CO_2_ and O_3_ under well-watered conditions. In the absence of any other treatment, water stress caused a decrease of the PI_total_, and it was partly eliminated by fumigation with elevated CO_2_ and CO_2_ + O_3_. This finding was also supported by the biomass results, which revealed a higher biomass under elevated CO_2_ and CO_2_ + O_3_. The decrease in biomass induced by elevated O_3_ was likely caused by the decline of photosynthetic efficiency. Our findings suggest that elevated CO_2_ reduces the drought effect both in the absence and presence of O_3_ in canola and wheat plants. The study also indicates that elevated O_3_ would pose a threat in future to agricultural crops.

## 1. Introduction

The concentrations of carbon dioxide (CO_2_) and ozone (O_3_) are increasing at a steady rate in the atmosphere [1,2]. The increasing CO_2_ trend is mostly caused by the use of fossil fuels for combustion [2]. It is projected that CO_2_ concentration will rise to about 550 ppm by 2050 [3]. Elevated CO_2_ stimulates plant growth and development but elevated O_3_ often has the opposite effect [4,5]. Ozone causes considerable damage in agricultural crops, which includes visible injury, reduced photosynthetic capacity, modifications to carbon allocation and reduced yield quantity and quality [6,7]. Prolonged exposure to O_3_ levels above 40 ppb decreases crop yields due to reduced photosynthesis and disruption of metabolism [8]. These findings suggest that agricultural crops in southern Africa may be at risk because of elevated O_3_ levels [9]. The seasonal variation of O_3_ indicates the highest O_3_ concentrations in spring and winter and the lowest in summer [10]. The maximum O_3_ concentrations in this region are between 40–60 ppb and can rise to more than 90 ppb in the spring season [11]. What is of concern is how these changes will interact with one another and influence plant growth, as well as the interaction of these gasses with other factors of climate change, such as droughts. 

Climate change will have effects on agriculture and food security [12]. Agriculture plays a vital role in the economy of developing countries. Subsistence farmers in the southern Africa region depend on staple crops for income generation. Commercial farmers will also be affected by climate change, which could affect food security on a local and global scale. The effect of elevated CO_2_ and O_3_ on important crops (and vegetation) has received a lot of attention in Europe and North America. To be specific, studies that relate to the effect of elevated CO_2_ in combination with O_3_ and droughts are very limited in southern Africa. As a result, the findings of developed countries have been extrapolated in developing countries [7], but given differences in climatic conditions, these results may not represent the local conditions. Plants grown under elevated CO_2_ have been shown to alleviate the effects of drought stress [13,14], due to CO_2_-induced increases in stomatal resistance. Moreover, elevated CO_2_ is understood to alleviate the harmful effects of O_3_ by reducing stomatal conductance and thus reducing O_3_ uptake and the potential for oxidant damage [15]. The combined effects of O_3_ with elevated CO_2_ and droughts are important but not well understood. In fact, there has been little consideration on how the different components of global climate change, such as O_3_, CO_2_, temperature and weather extremes, might combine and interact to influence the agricultural sector [7]. 

Photosynthesis is regarded as a reliable measure of the overall performance in plants [16]. It is one of the important processes to be affected by abiotic stress, such as elevated O_3_ and droughts, which cause a decline in CO_2_ diffusion to the chloroplast and metabolic constraints [17]. Drought stress gradually decreases CO_2_ assimilation rates because of reduced stomatal conductance [18]. Stomatal closure is the initial response to droughts and the main limitation of photosynthesis at mild to moderate drought stress [19]. At a time of severe drought, the inhibition of metabolic process induces reduction in the contents of ribulose bisphosphate, which becomes the main limitation and thus inhibits photosynthetic CO_2_ assimilation [19]. In addition, drought stress enhances the generation of active oxygen species and antioxidant defenses [18]. In a meta-analysis of photosynthesis and drought, Pinheiro and Chaves [20] indicated the interaction of sugars, reactive oxygen species and hormones with photosynthetic responses to droughts. Furthermore, Zivcak et al. [21] showed that under drought stress conditions there is a complex interconnected and regulated photoprotective response.

In order to study the effects of elevated CO_2_ and O_3_ on the photosynthetic apparatus of canola and wheat plants, the chlorophyll (Chl) *a* fluorescence transients OJIP (a non-destructive, simple and rapid technique for sensing stress) was applied. Analysis of the OJIP transients by the JIP-test provides significant information about the structure and function of the photosynthetic apparatus [22,23,24]. The parameters calculated by the JIP-test and the shape of the transients have been found to be very sensitive to stress caused by environmental conditions, such as light intensity, temperature, drought, flooding, atmospheric CO_2_ or elevated O_3_ and chemical influences [25,26,27,28,29]. Therefore, it was hypothesized that elevated CO_2_ reduces the water stress (water deficit) effect both in the absence and presence of O_3_ in canola and wheat crops. The hypothesis was tested by exposing canola and wheat plants to elevated levels of CO_2_, O_3_ and the combination of these two gases in open-top chambers (OTCs) under well-watered and water-stressed (water deficit) conditions to quantify the biophysical and physiological responses. The objective was to understand how locally cultivated canola and wheat plants respond to elevated CO_2_ and O_3_ and if these environmental factors will interact with each other under well-watered and water-stressed conditions. 

## 2. Results

### 2.1. Photosynthetic Responses of Canola to Elevated Levels of CO_2_ and O_3_

**Figure 1:**
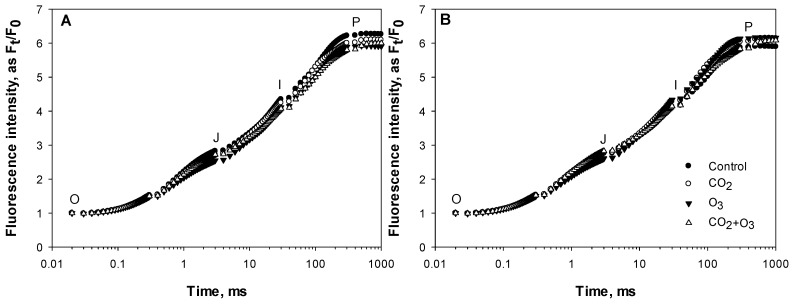
Average (of all weeks) chlorophyll (Chl) *a* fluorescence transients of canola leaves exposed to elevated CO_2_, O_3_ and CO_2_ + O_3_ under well-watered (**A**) and water-stressed conditions (**B**).

**Figure 2:**
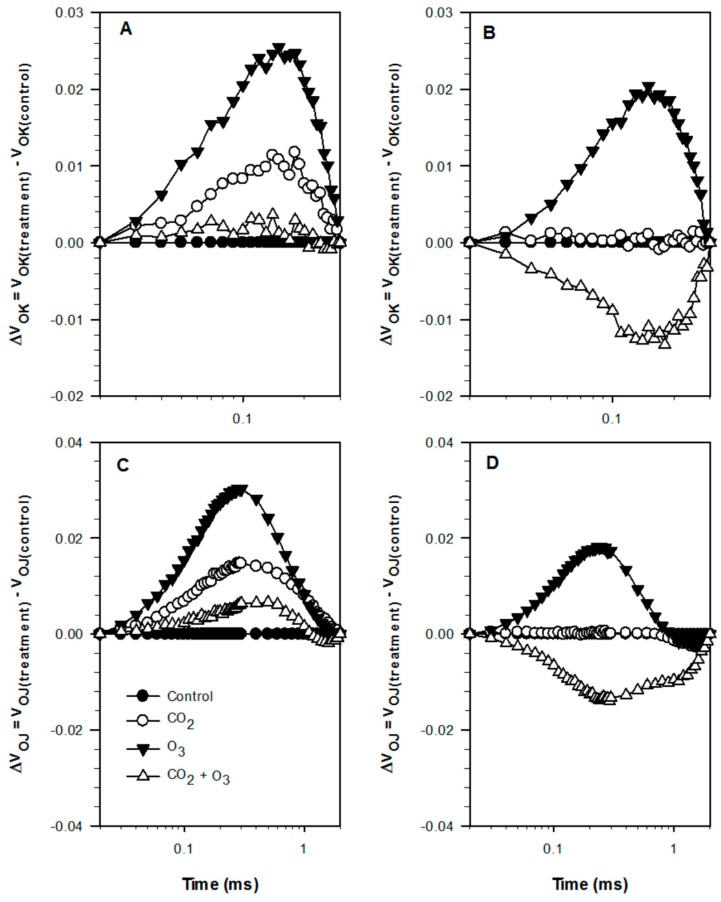
Effect of elevated CO_2_, O_3_ and the combination of CO_2_ and O_3_ on differential plots of relative chlorophyll a fluorescence (ΔV_t_) under well-watered (**B**,**D**) and water-stressed conditions (**A**,**C**) in leaves of canola. The data represent the average of all weeks. (**A**,**B**), ΔV_OK_ = V_OK_ (treatment) − V_OK (control)_; (**C**,**D**), ΔV_OJ_ = V_OJ (treatment)_ − V_OJ (control)_.

The averages of the raw fluorescence transients of canola leaves were plotted on a logarithmic time scale from 20 µs to 1 s and the values are expressed as F_t_/F_0_ (Figure 1). The steps O, J, I and P are indicated. In order to reveal hidden differences, the fluorescence data were normalized between O (20 μs) and K (300 μs) steps, as V_OK_ = (F_t_ − F_0_)/(F_K_ − F_0_), and plotted as difference kinetics ΔV_OK_ = V_OK(treatment)_ − V_OK (control)_. Also, fluorescence data were normalized between the steps O and J (2 ms), as V_OJ_ = (F_t_ − F_0_)/(F_J_ − F_0_), and plotted as difference kinetics ΔV_OJ_ = V_OJ (treatment)_ − V_OJ (control)_. These allowed the visualization of the positive ΔL (0.15 ms) and ΔK-band (0.3 ms) in the O_3_ treatment (Figure 2). The appearance of ΔL and ΔK-band is regarded as a good indicator to detect the physiological disturbances of plants caused by environmental conditions [30].

**Figure 3:**
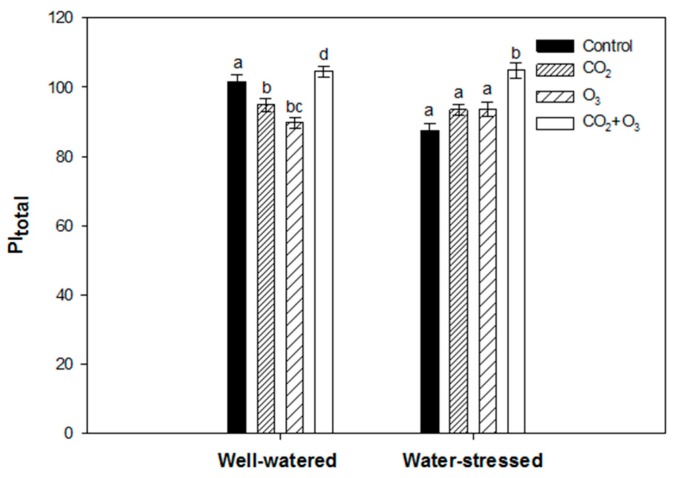
Average (of all weeks) PI_total_ of canola plants exposed to CO_2_, O_3_ and CO_2_ + O_3_ under well-watered and water-stressed conditions for four weeks. For the same water treatment, different letters show statistically significant differences (*p* < 0.05).

Canola plants fumigated with elevated CO_2_ and O_3_ caused the decline of the PI_total_ when subjected to well-watered conditions (Figure 3). However, in water-stressed plants, no significant difference was found when compared with the control. The combination of elevated CO_2_ and O_3_ had higher PI_total_ values under both water regimes (Figure 3).

The PI_total_ is an overall parameter calculated from the JIP-test that combines biophysical parameters. The parameters are the density of reaction centers (RC/ABS); the parameter (*φ*_Po_ /(1 − *φ*_Po_), where *φ*_Po_ represents the maximum quantum yield of primary photochemistry; the parameter (*ψ*_Eo_ /(1 − *ψ*_Eo_), where *ψ*_Eo_ represents the efficiency with which an electron moves into the electron transport chain further than Q_A_^-^; the parameter (*δ*_Ro_ /(1 − *δ*_Ro_), where *δ*_Ro_ represents the efficiency with which an electron from the intersystem electron carriers is transferred to reduce end electron acceptors at the PSI (Photosystem I) acceptor side. The statistical analysis of the effect of elevated CO_2_, O_3_ and CO_2_ + O_3_ on the components of the PI_total_ in canola leaves is shown in Table 1. 

Plants in the O_3_ treatment enhanced the parameter *ψ*_Eo_/(1 − *ψ*_Eo_) under both water regimes, while the parameter *δ*_Ro_ /(1 − *δ*_Ro_) was reduced. The combination of CO_2_ and O_3_ improved the parameter *δ*_Ro_ /(1 − *δ*_Ro_) when subjected to well-watered and water-stressed conditions (Table 1).

**Figure 4:**
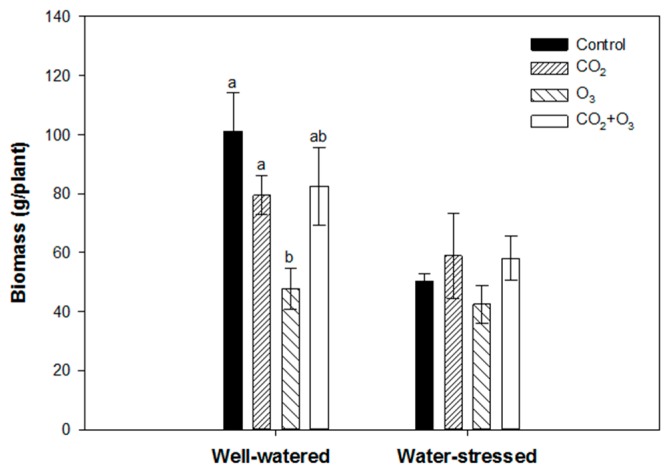
Biomass of canola exposed to CO_2_, O_3_ and CO_2_ + O_3_ under well-watered and water-stressed conditions after four weeks. For the same treatment, different letters show statistically significant differences (*p* < 0.05).

Elevated CO_2_ and the combination of CO_2_ and O_3_ had no significant effect on the above ground biomass of well-watered plants. However, canola plants exposed to O_3_ (80 ppb) caused a significant reduction in biomass under well-watered conditions (Figure 4). In water-stressed plants, none of the treatments had a significant effect on biomass production, but based on average values, elevated CO_2_ and the combination of CO_2_ and O_3_ led to a higher biomass than the control (Figure 4).

**Figure 5:**
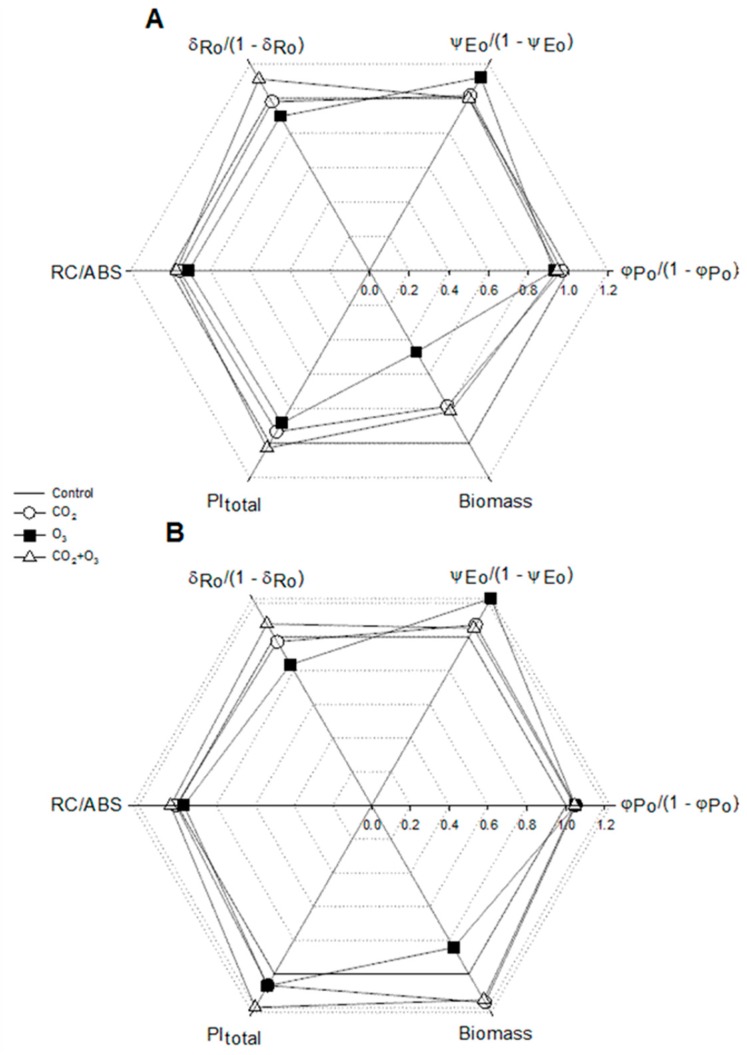
Radar plot of selected biophysical parameters (PI_total_, RC/ABS, *φ*_Po_ /(1 − *φ*_Po_), *ψ*_Eo_ /(1 − *ψ*_Eo_), *δ*_Ro_ /(1 − *δ*_Ro_)) and biomass for canola. Values were normalized on those of the control, which is presented by the regular hexagon for well-watered (**A**) and water-stressed (**B**) plants.

The radar plot indicates that the decline of PI_total_ in well-watered canola plants exposed to elevated O_3_ was caused by the parameter *δ*_Ro_ /(1 − *δ*_Ro_) (Figure 5). This drop was linked to the decrease of biomass accumulation. The higher PI_total_ in canola plants fumigated with a combination of CO_2_ and O_3_ was sustained by the parameter, *δ*_Ro_ /(1 − *δ*_Ro_).

### 2.2. Photosynthetic Responses of Wheat to Elevated Levels of CO_2_ and O_3_

**Figure 6:**
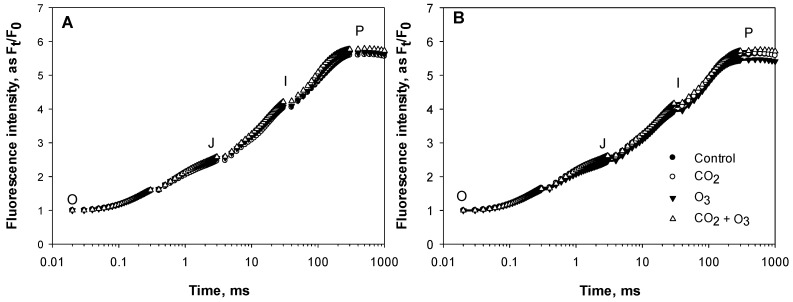
Average (of all weeks) Chl *a* fluorescence transients of dark-adapted wheat leaves exposed to elevated CO_2_, O_3_ and CO_2_+O_3_ under well-watered (**A**) and water-stressed (**B**) conditions.

The average Chl *a* fluorescence transient of dark-adapted wheat leaves for the four treatments under well-watered (Figure 6A) and water-stressed (Figure 6B) conditions were plotted on a logarithmic time scale, 20 μs to 1 s and expressed as F_t_/F_0_ for clarity. The four transients showed a typical OJIP shape, with small differences between them. In order to show the hidden differences, the fluorescence data were normalized between O (20 μs) and K (300 μs) steps, as V_OK_ = (F_t_ − F_0_)/(F_K_ − F_0_), and plotted as difference kinetics ΔV_OK_ = V_OK(treatment)_ − V_OK (control)_, which revealed the ΔL-band. A positive ΔL-band indicates lower energetic connectivity while a negative ΔL-band indicates higher energetic connectivity [31]. Furthermore, the fluorescence data were normalized between the steps O and J (2 ms), as V_OJ_ = (F_t_ − F_0_)/(F_J_ − F_0_), and plotted as difference kinetics ΔV_OJ_ = V_OJ(treatment)_ − V_OJ (control)_ revealing the ΔK-band. 

**Figure 7:**
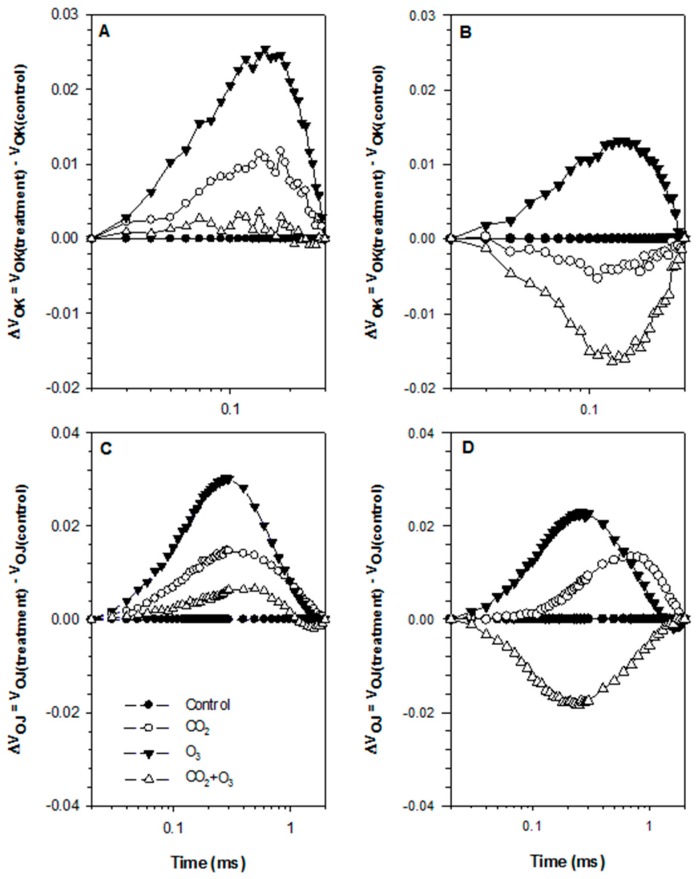
The effect of elevated CO_2_, O_3_ and CO_2_ +O_3_ on differential plots of relative Chl *a* fluorescence (ΔV_t_) under well-watered (**A**,**C**) and water-stressed conditions (**B**,**D**) in leaves of wheat. The data represent the average of all weeks. (**A**,**B**), ΔV_OK_ = V_OK (treatment)_ − V_OK (control)_; (**C**,**D**), ΔV_OJ_ = V_OJ (treatment)_ − V_OJ (control)_.

A positive ΔK-band shows an increased reduction rate of quinone (Q_A_), from Q_A_ to Q_A_^−^, which suggests that the oxygen evolving complex (OEC) may have become leaky and offers access to non-water electron donors [32]. Positive ΔL- and ΔK-bands in the O_3_ treatment were revealed clearly under both water regimes (Figure 7).

**Figure 8:**
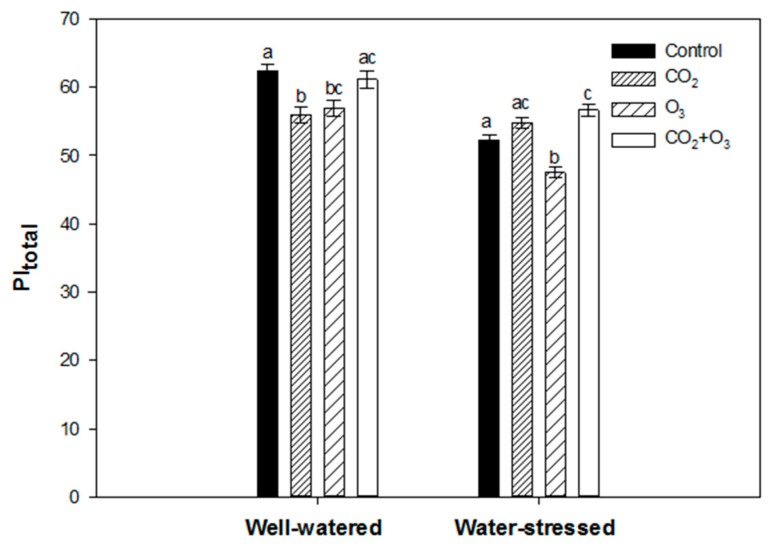
Average (of all weeks) PI_total_ of wheat plants exposed to CO_2_, O_3_ and CO_2_+O_3_ under well-watered and water-stressed conditions for four weeks. For the same water treatment, the different letters show statistically significant differences (*p* < 0.05).

Elevated CO_2_ resulted in a significant decrease of the total performance index (PI_total_) in wheat plants subjected to well-watered conditions (Figure 8). However, no significant difference was found in water-stressed plants exposed to elevated CO_2_. Ozone fumigation led to a significant decline in PI_total_ under both water regimes. The combination of elevated CO_2_ and O_3_ did not affect the photosynthetic performance (based on the PI_total_) of well-watered wheat plants. However, in water-stressed wheat plants, the PI_total_ increased significantly by 9%, compared with the non-fumigated plants (Figure 8).

The statistical analysis of the effect of elevated CO_2_, O_3_ and CO_2_ + O_3_ on these parameters under well-watered and water-stressed conditions is presented in Table 2. We note that fumigation on wheat plants had a significant effect on the components of the PI_total_ with the exception of the parameter (*ψ*_Eo_ /(1 − *ψ*_Eo_) in well-watered plants. Elevated CO_2_ and O_3_ caused a significant decline in the density of reaction centers and the parameter *δ*_Ro_ /(1 − *δ*_Ro_) (Table 2). The combination of CO_2_ and O_3_ enhanced the density of reaction centers under both water regimes.

**Figure 9:**
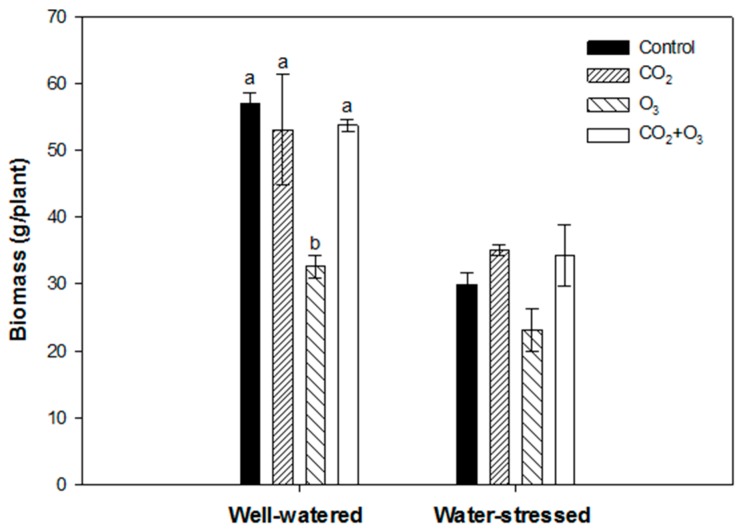
Biomass of wheat exposed to CO_2_, O_3_ and CO_2_+O_3_ under well-watered and water-stressed conditions after four weeks. For the same water treatment, the different letters show statistically significant differences (*p* < 0.05).

Ozone fumigation caused a reduction in biomass of wheat plants when subjected to well-watered and water-stressed conditions. Compared with the control, biomass was reduced by about 40% under well-watered and 22% under water-stressed conditions (Figure 9). In water-stressed plants, fumigation did not have any significant effect on biomass. However, based on average values the plants exposed to elevated CO_2_ and CO_2_ + O_3_, treatments had a higher biomass compared with the control (Figure 9). 

**Figure 10:**
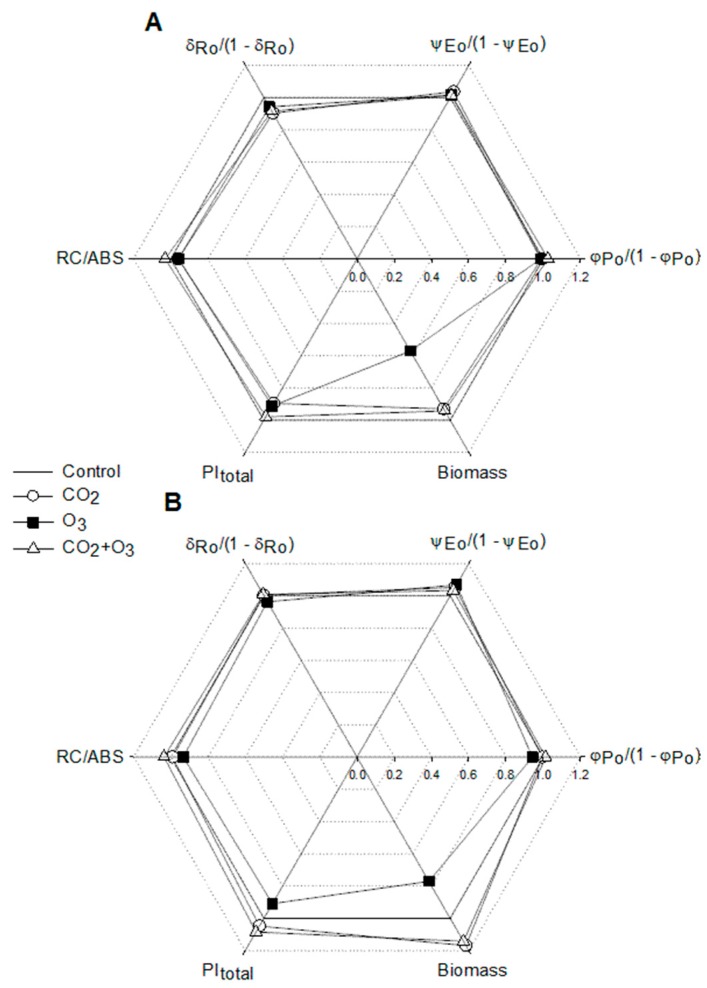
The radar plot of selected biophysical parameters (PI_total_, RC/ABS, *φ*_Po_ /(1 − *φ*_Po_), *ψ*_Eo_ /(1 − *ψ*_Eo_), *δ*_Ro_ /(1 − *δ*_Ro_) and biomass of wheat. Values were normalized on those of the control, which is shown by the regular hexagon for well-watered (**A**) and water-stressed (**B**) plants. For the JIP-test parameters, the data represent the average of all weeks.

The radar plot of the PI_total_ and its components and biomass is presented in Figure 10. As shown, the reduction in biomass of ozone-treated plants is related to the decrease of the PI_total._ On the other hand, the biomass enhancement in water-stressed wheat plants exposed to the combined effect of elevated CO_2_ and O_3_ is well associated with the increase in PI_total_. We noted that the PI_total_ was generally influenced by the density of reaction centers and efficiency, with which an electron from the intersystem electron carriers is transferred to reduce end electron acceptors at the PSI acceptor side (Figure 10)_._

## 3. Discussion

Elevated CO_2_ increases the photosynthetic performance of plants, which in turn results in higher biomass production [33,34], but often declines with time when plants are subjected to elevated CO_2_ over extended periods [33]. Prolonged exposure of plants to elevated CO_2_ reduces the initial stimulation of photosynthesis [35] and as a result suppresses photosynthesis, which reduces growth responses [36]. It has been suggested that crop plants subjected to reduced water will respond positively to elevated CO_2_ in comparison to crop plants under sufficient water supply, as CO_2_ causes an increase in stomatal resistance [5]. We found that plants fumigated with elevated CO_2_ resulted in a decline of the photosynthetic performance (as revealed by the PI_total_) under well-watered conditions in both crop species. The decline of the PI_total_ can be ascribed to exposing the plants to elevated CO_2_ for more than four weeks. The decrease in the PI_total_ under well-watered conditions was mainly influenced by the density of reaction centers and efficiency with which an electron from the intersystem electron carriers is transferred to reduce end electron acceptors at the PSI acceptor side (*δ*_Ro_). A low I-P amplitude may indicate a low capacity of electron transport through PSI. It was shown that the activity of PSI can limit photosynthetic electron transport through PSI, and that PSI can be limiting for CO_2_ assimilation [37,38], which can be especially important under elevated CO_2_ [39]. Liu et al. [40] reported that elevated CO_2_ enhanced the photosynthetic performance of cucumber plants under moderate drought stress. We found that none of the water-stressed plants were affected by elevated CO_2_, but on average the PI_total_ values were higher than those of the control. The PI_total_ values were well-maintained by an increase of the parameter *δ*_Ro_, indicating an increased capacity to reduce end acceptors beyond PSI. The increase of the I-P amplitude was shown to be associated also with an increase of capacity in alternative electron transport pathways [41]. In addition, above ground biomass in both crops was not significantly affected by elevated CO_2_ when subjected to well-watered and water-stressed conditions. However, previous studies reported that elevated CO_2_ significantly increased biomass in canola and wheat plants [42,43,44]. The difference can be attributed to the decline in the photosynthetic capacity of the plants, as indicated by the PI_total_, and that the response of crops to elevated CO_2_ varies between genotypes [5]. Even though there was no significant difference in the water-stressed plants, the CO_2_ treatment enhanced the biomass of both crops. The biomass stimulation caused by elevated CO_2_ can also result from reduced water loss and water stress, and/or from decreased respiration [45]. The greater biomass in dry conditions in response to elevated CO_2_ has been discussed by Fitzgerald et al. [46]. It should be noted that the beneficial effect of elevated CO_2_ in plants subjected to water-stress not only increases biomass production, but also translates into higher crop yield [14,47]. Elevated CO_2_ stimulated yield of water-stressed wheat plants (data not shown). Our results suggest that elevated CO_2_ concentrations may counteract the negative effect of droughts on canola and wheat. Therefore, it appears that canola and wheat crops grown in limited water availability will benefit more from elevated CO_2._

The appearance of the positive ΔL- and ΔK-bands indicates that both crops are more sensitive to elevated O_3_ with reference to lower energetic connectivity and inactivation of the OEC, respectively [26,31,48]. Similar findings were reported by Desotgiu et al. [49] in poplar plants subjected to O_3_ and water stress. The amplitude of the ΔK-band was higher in the well-watered plants when compared with the water-stressed plants. This suggests that limited water availability can reduce inactivation of the OEC. The OEC represents one of the sensitive components of the PSII (Photosystem II) [50,51]. Furthermore, the appearance of the K-band revealed that elevated O_3_ upset the functioning of OEC in the PSII [52]. The transients were further analyzed with the JIP-test equations, which led to the calculation of several photosynthetic parameters and the PI_total_. In the present experiment, both crop species—canola and wheat—showed a tendency to reduce the PI_total_ in the O_3_ treatment (80 ppb) particularly under well-watered conditions. Under drought conditions, the decrease was significant only in wheat plants. This indicates that the effect of O_3_ was minor under drought conditions in canola plants [53]. The reduction of the PI_total_ was caused by the efficiency with which an electron from the intersystem electron carriers was transferred to reduce end electron acceptors at the PSI acceptor side, as well as the decline of the density of reaction centers, which, taking into account that *φ*_Po_ remained nearly unchanged, represented an increase of the functional PSII antenna size [26]. The efficiency that an electron moves further than Q_A¯_ was relatively less affected by elevated O_3_ in both plants. Although a temporarily enhanced *ψ*_Eo_ was detected in canola plants, it did not influence the PI_total_. The increased efficiency was linked to the activating of repair processes, but when it was linked to a reduced end acceptor capacity in combination with a reduced Calvin cycle, energy demand led to over-excitation of the photosynthetic apparatus [27]. These results support the findings obtained from other studies that the I-P region (as revealed by the relevant parameters) is sensitive to stress caused by O_3_ [27,54]. The biomass was significantly affected by O_3_ fumigation in both crop plants under well-watered conditions. There was no significant difference under water-stressed treatment, but based on average values, the biomass was reduced in O_3_ treatment. In the experiment, where fumigation with 60 ppb of O_3_ was applied in canola, the biomass was barely affected [43], and similar findings were reported in a study using four canola cultivars [44]. On the contrary, Feng et al. [55] found that elevated O_3_ decreased above ground biomass by 18%. The response to O_3_ achieved in the present study can be interpreted by the higher than 60 ppb concentration applied, and to cultivar differences [56]. Similarly, only two out of five wheat cultivars showed a decrease in above ground biomass when subjected to O_3_ [57]_._ Based on the radar, it was suggested that the drop in biomass production was associated with the decline of the PI_total_ [58,59]. 

The ΔL-band exhibited differences in energetic connectivity among the PSII units [26]. The energetic connectivity among PSII units improved in the CO_2_ + O_3_ treatment under water-stressed conditions. This was demonstrated by the appearance of the negative ΔL-band, which indicated higher energetic connectivity. A higher energetic connectivity resulted in an improved use of the excitation energy and a higher stability of the photosynthetic system [31,60]. This suggests that elevated CO_2_ and reduced water supply could improve the utilization of excitation energy and the stability of photosynthetic systems. Furthermore, the appearance of the negative ΔK-band under water stress showed that plants fumigated with elevated CO_2_ + O_3_ have either a more active oxygen evolving system or a smaller PSII antenna size [60]. These effects should be regarded as favorable for the photosynthetic apparatus [60]. As shown by the PI_total_, fumigation with elevated CO_2_ + O_3_ increased the photosynthetic efficiency of canola plants under both well-watered and water-stressed conditions. In wheat plants, the increase was significant only under water-stressed conditions. The increase in the PI_total_ was mostly caused by the parameter *δ*_Ro_. The combinations of CO_2_ and O_3_ did not reveal any significant reductions in biomass. This suggests that elevated CO_2_ can ameliorate the detrimental effects of elevated O_3_ and droughts upon canola and wheat. In addition, the photosynthetic process was not compromised as a result of the combined effects of elevated CO_2_ and O_3_. The increase in photosynthetic performance (as revealed by the JIP-test parameters) was associated with the increase in biomass production. This indicates that biomass enhancement was most probably caused by the increase in photosynthetic efficiency of the plants. 

## 4. Materials and Methods 

### 4.1. Experimental Site and Plant Materials

The experiments were conducted in Open-Top Chambers situated at the North-West University, South Africa. The canola and wheat experiments were performed from June to August in 2014 and 2015, respectively. Heyneke et al. [61] has discussed the design and operation of the OTCs system used in the current study. Canola (*Brassica napus* L. cv. Rainbow) and wheat (*Triticum aestivum* L. cv. SST875) seeds were sown in pots with a diameter of about 30 cm. The pots were watered manually prior to the start of fumigation with elevated CO_2_ and O_3_ to ensure that the seeds germinated successfully. Six-month slow release fertilizer (25 g) was added to each pot comprising 17 nitrogen:11 phosphorus:10 potassium:2 magnesium oxide:TE (Osmocote Pro, The Netherlands). The growth medium was composed of topsoil, river sand and vermiculite (2:1:1). The pots were placed into eight OTCs. Two chambers were used per treatment. The treatments were the control (carbon filtered air, OTC 1 and 2); CO_2_ (700 ppm, OTC 3 and 4); O_3_ (80 ppb, OTC 5 and 6) and CO_2_ + O_3_ (700 ppm + 80 ppb, OTC 7 and 8). The carbon filtered air was used only for the control treatment, from which O_3_ and other pollutants were removed. 

### 4.2. Fumigation and Water Treatment 

Plants were exposed to elevated CO_2_ (700 ppm) and O_3_ (80 ppb) and the combination of these two gases (CO_2_ + O_3_) from 08:00am to 17:00pm for 4 weeks. Elevated CO_2_ levels inside the OTCs were monitored with a CO_2_ monitor (Model 174687 CO_2__temp-relative-humidity monitor, Scientific Associates, Inc., China). Ozone levels were continuously monitored using an O_3_ monitor (Model 205 Ozone Monitor, 2B Technologies, Inc., USA). The temperatures varied between 23 °C and 17 °C during the fumigation period. The ambient ozone levels were below 35 ppb over the entire fumigation period. 

The plants were exposed to two water regimes, namely water-watered and water-stressed (water deficit) conditions. All the plants absorbed water through glass fiber wicks that were projected into water reservoirs. In the well-watered treatment, four wicks were placed at four levels within the pots, while in the water-stressed treatment, one glass fiber wick was positioned at the middle level of each pot [43]. The pots were positioned into reservoirs that were connected to a drip irrigation system that filled up the water. 

### 4.3. Chlorophyll a Fluorescence

The fast chlorophyll *a* fluorescence transients were measured with a Handy PEA (Plant Efficiency Analyser) fluorimeter (Hansatech Instruments Ltd, UK) on the leaves of canola and wheat. Before measurements were taken, the leaves were dark-adapted for an hour. The OJIP transients were induced by red light (peak at 650 nm) of 3000 μmol photons m^−2^ s^−1^ provided by an array of three light-emitting diodes and recorded for 1 s with 12 bit resolution. The data acquisition occurred at every 10 μs, from 10 μs to 0.3 ms; every 0.1 ms, from 0.3 to 3 ms; every 1 ms, from 3 to 30 ms; every 10 ms, from 30 to 300 ms and every 100 ms, from 300 ms to 1 s. The OJIP transients were analyzed by the JIP-test [19,50] using the PEA Plus ver. 1.10 Program (Hansatech Instruments Ltd, UK). The following fluorescence data from the original measurements were used by the JIP-test: the minimal intensity at 20 µs (O step); the intensities at 50 and 300 µs (F_300_ and F_50_) used for calculation of the initial slope (M_0_); the intensity at 2 ms (J step); the intensity at 30 ms (I step) and the maximal measured intensity when all PSII reaction centers (RCs) were closed (F_m_, P step). The following JIP-test parameters were derived from the OJIP transients, all referring to time zero (onset of fluorescence induction): the maximum quantum yield of primary photochemistry, *φ*_Po_ = TR_0_/ABS = [1 − (F_0_/F_M_)]; the efficiency/probability that an electron moves further than Q_A_^−^ into the electron transport chain, *ψ*_Eo_ = ET_0_/TR_0_ = (1 − V_J_); the efficiency/probability with which an electron from the intersystem electron carriers is transferred to reduce end electron acceptors at the PSI acceptor side (RE), *δ*_Ro_ = RE_0_/ET_0_ = (1 − V_I_)/(1 − V_J_); the density of reaction centers, RC/ABS = γ_RC_ /(1 − γ_RC_) = *φ*_Po_ (V_J_/M_0_) and the total performance index (PI_total_) = [γ_RC_ /(1 − γ_RC_)], [*φ*_Po_ /(1− *φ*_Po_)], [*ψ*_Eo_ /(1 − *ψ*_Eo_)], [*δ*_Ro_ /(1 − *δ*_Ro_)], an index (potential) for energy conservation from excitons to the reduction of PSI end-electron acceptors.

### 4.4. Biomass

Plants were harvested after four weeks and the fresh plant material was oven-dried at 60 °C for 72 h and weighed.

### 4.5. Statistical Analysis

Statistical analysis were carried out with STATISTICA 13 (Stat Soft. Inc., Tulsa, OK, USA). 

The data were analyzed using one-way analysis of variance (ANOVA) and significant differences between treatment means were determined by the Tukey’s honest significant difference (HSD) post-hoc test. 

## 5. Conclusions

In conclusion, elevated O_3_ led to a decrease in biomass of canola and wheat plants. This reduction was caused by a decline in the photosynthetic efficiency as revealed by the total performance index (PI_total_). The results of the current study indicate that elevated O_3_ would pose a threat in the future to agricultural crops. The decline in the PI_total_ was mostly influenced by the efficiency with which an electron from the intersystem electron carriers was transferred to reduce end electron acceptors at the PSI acceptor side. The present study also suggests that the PSII was damaged and the photosynthetic apparatus was compromised due to elevated O_3_. Elevated CO_2_ reduces the drought effect both in the absence and presence of O_3_. This was also supported by above ground biomass results, which showed higher values under elevated CO_2_. Our findings suggest that elevated CO_2_ can reduce the negative effect of abiotic stress, such as droughts and O_3_ in canola and wheat plants. The measurement of Chl *a* fluorescence can be used to screen the effect of elevated CO_2_ and O_3_ in canola and wheat plants cultivated locally. Further studies should seek to investigate several local varieties of canola and wheat responses to elevated CO_2_, O_3_ and droughts and their interaction. 

## Figures and Tables

**Table 1:** The effect of elevated CO_2_, O_3_ and CO_2_ + O_3_ on the components of the PI_total_ in canola plants under well-watered and water-stressed conditions. Values are means of weeks and SE (Standard Error). Different letters in the same row indicate statistically significant differences between the treatments (*p* < 0.05).

**Well-Watered**	**Control**	**CO_2_**	**O_3_**	**CO_2_ + O3**	***p*-level**
*φ*_Po_ /(1 - *φ*_Po_)	5.775 ± 0.033a	5.584 ± 0.029b	5.359 ± 0.027c	5.459 ± 0.031c	<0.001
*ψ*_Eo_ /(1 - *ψ*_Eo_)	2.181 ± 0.043a	2.214 ± 0.029a	2.444 ± 0.031b	2.180 ± 0.032a	<0.001
*δ*_Ro_ /(1 - *δ*_Ro_)	1.164 ± 0.019a	1.140 ± 0.023a	1.042 ± 0.011b	1.293 ± 0.020c	<0.001
Density of reaction centers (RC/ABS)	0.725 ± 0.005a	0.692 ± 0.004b	0.659 ± 0.004c	0.704 ± 0.004b	<0.001
**Water-Stressed**					
*φ*_Po_ /(1 - *φ*_Po_)	5.388 ± 0.038a	5.652 ± 0.036b	5.643 ± 0.025b	5.624 ± 0.036b	<0.001
*ψ*_Eo_ /(1 - *ψ*_Eo_)	2.020 ± 0.034a	2.164 ± 0.033b	2.480 ± 0.027c	2.124 ± 0.035ab	<0.001
*δ*_Ro_ /(1 - *δ*_Ro_)	1.154 ± 0.016a	1.121 ± 0.016a	0.964 ± 0.012b	1.243 ± 0.021c	<0.001
RC/ABS	0.695 ± 0.004a	0.699 ± 0.004a	0.671 ± 0.005b	0.718 ± 0.005c	<0.001

**Table 2:** The effect of elevated CO_2_, O_3_ and CO_2_ + O_3_ on the components of the PI_total_ in wheat plants under well-watered and water-stressed conditions. Values are means of weeks and SE. Different letters in the same row indicate statistically significant differences between the treatments (*p* < 0.05).

**Well-Watered**	**Control**	**CO_2_**	**O_3_**	**CO_2_ + O_3_**	***p*-level**
*φ*_Po_ /(1 - *φ*_Po_)	5.034 ± 0.021a	4.987 ± 0.030a	4.965 ± 0.22a	5.161 ± 0.033b	<0.001
*ψ*_Eo_ /(1 - *ψ*_Eo_)	2.321 ± 0.036a	2.406 ± 0.039a	2.357 ± 0.033a	2.354 ± 0.043a	N.S.
*δ*_Ro_ /(1 - *δ*_Ro_)	0.974 ± 0.010a	0.880 ± 0.014b	0.918 ± 0.015b	0.892 ± 0.016b	<0.001
RC/ABS	0.555± 0.004a	0.533 ± 0.003b	0.532 ± 0.003b	0.572 ± 0.004c	<0.001
**Water-Stressed**					
*φ*_Po_ /(1 - *φ*_Po_)	5.039 ± 0.022a	5.013 ± 0.021a	4.745 ± 0.028b	5.101 ± 0.028a	<0.001
*ψ*_Eo_ /(1 - *ψ*_Eo_)	2.125 ± 0.030a	2.236 ± 0.025b	2.270 ± 0.031b	2.196 ± 0.037ab	0.006
*δ*_Ro_ /(1 - *δ*_Ro_)	0.948 ± 0.011ab	0.952 ± 0.009a	0.913 ± 0.010b	0.956 ± 0.015a	0.017
RC/ABS	0.520 ± 0.003a	0.515 ± 0.002a	0.485 ± 0.003b	0.539 ± 0.003c	<0.001
